# The effects of aromatase inhibitors on lipids and thrombosis

**DOI:** 10.1038/sj.bjc.6602692

**Published:** 2005-08-15

**Authors:** N J Bundred

**Affiliations:** 1 South Manchester University Hospital, Academic Surgery, Education and Research Centre, Southmoor Road, Manchester M23 9LT, UK

**Keywords:** tamoxifen, aromatase inhibitors, lipids, thromboembolic events

## Abstract

Oestrogen is known to influence blood lipid levels and though its cardioprotective effects are less clear than once thought, there remains concern that reduction of oestrogen levels during hormonal treatment for breast cancer may have an adverse effect on cardiovascular risk. While tamoxifen has been shown to improve lipid profiles, the aromatase inhibitors have a very different mode of action and do not possess the oestrogen-agonistic effects of tamoxifen. At present, there are few data on the effects of these agents on lipid profiles. Available data are mixed, but suggest that the different aromatase inhibitors have different effects on lipid profiles. Some studies show anastrozole as generally having little effect on lipids, while others have indicated adverse effects on lipid profiles/increased hypercholesterolaemia. Letrozole has been associated with adverse effects on lipid profiles in some studies, including BIG 1-98, but short-term data from randomised trials do not show increased cardiovascular morbidity. By contrast, exemestane, which has been studied in slightly more detail, may either have little effect or may be associated with slightly improved lipid profiles. In general, the changes have been small and are likely to be of little relevance in women with advanced breast cancer, but if these agents come to be used in early breast cancer, their impact on lipid profiles may become more important. Many studies are currently underway with the aromatase inhibitors, with safety assessments including monitoring lipid levels. The results of these studies are keenly awaited.

Clinical evidence points to low-density lipoprotein (LDL) cholesterol as the key factor in the pathogenesis of atherosclerosis and coronary heart disease (CHD), while high-density lipoprotein (HDL) cholesterol appears to have a protective effect. Increases in LDL cholesterol are associated with increased risk for CHD, even when total cholesterol concentration is within the normal range. Women generally appear to have higher levels of HDL cholesterol than men. Furthermore, some studies have suggested that low levels of HDL cholesterol, particularly high total/HDL ratio or low HDL_2_ subfraction, could be a more significant risk factor for women than men, with total cholesterol being less important for women (Kannel, 1987; Miller-Bass *et al*, 1993). The role of triglycerides is controversial, but elevated triglycerides have been suggested to be a stronger predictor of cardiovascular diseases in women than in men (Korhonen *et al*, 1996; La Rosa, 1997). Finally, high lipoprotein (a) (Lp(a)) levels have been associated with an increased risk both in women and men (Danesh *et al*, 2000).

Oestrogen is known to affect blood lipids, with levels of HDL cholesterol increasing and LDL cholesterol decreasing with rising levels of oestrogen. Reduced oestrogen levels, such as occurs at menopause, has been found to cause atherogenic alterations in lipid and lipoprotein profiles in epidemiological studies comparing premenopausal women with menopausal and postmenopausal women (Schaefer *et al*, 1994; de Aloysio *et al*, 1999; Tremollieres *et al*, 1999), and also in longitudinal trials (Do *et al*, 2000). Increases in both total and LDL cholesterol concentrations are seen during menopause. Additionally, a shift to smaller, denser and potentially more atherogenic LDL particle sizes has been noted (Campos *et al*, 1988). Data on HDL cholesterol have been inconsistent, as HDL cholesterol has been reported to remain unaffected in some studies (Kannel, 1987), while others have noted a decline of HDL cholesterol, particularly HDL_2_ subfraction, with a rise of HDL_3_ cholesterol, which may result in an unfavourable net effect (Matthews *et al*, 1994). Increases in plasma triglycerides and Lp(a) concentrations have also been reported after menopause (Stevenson *et al*, 1993).

However, there has been controversy about the importance of endogenous oestrogens to cardiovascular risk, because age and menopause are closely related and lipid profiles change with age. In fact, the influence of menopause on lipoprotein changes varies between different studies (Stevenson *et al*, 1993), and its influence has been suggested to be lower when age and other confounding factors are taken into account (Matthews *et al*, 1994; de Aloysio *et al*, 1999).

For many years it was accepted that hormone replacement therapy with oestrogen could partly reverse the increase in cardiovascular risk occurring at menopause. However, the cardioprotective effects of exogenous oestrogen have now been called into question (Hulley *et al*, 1998; Women's Health Initiative Steering Committee, 2004). Despite doubts surrounding the effects of oestrogen on cardiovascular risk, there remains concern that lowering oestrogen levels during adjuvant therapy for breast cancer might have detrimental effects on lipid levels and might also increase cardiovascular risk. This is particularly relevant given that the greatest cause of death in women with early stage breast cancer is heart disease (Levi *et al*, 2002). Although tamoxifen has not been shown to increase the risk of cardiovascular disease, it has a very different mode of action from the aromatase inhibitors, which are increasingly being used in the adjuvant setting. This has triggered considerable interest in the lipid effects of these agents.

## EFFECTS OF ANTI-OESTROGEN TREATMENT ON LIPID PROFILES

### Tamoxifen

Tamoxifen has oestrogenic agonist effects on blood lipids, with several studies describing small changes in plasma lipoprotein concentrations, most of the changes potentially reducing the risk of cardiovascular disease (Dziewulska-Bokiniec *et al*, 1994; Thangaraju *et al*, 1994; Love *et al*, 1994; Grey *et al*, 1995; Decensi *et al*, 1998; Vrbanec *et al*, 1998; Cushman *et al*, 2001). Total and LDL cholesterol levels are decreased (Grey *et al*, 1995) and there is a tendency for HDL cholesterol levels to be increased, although this is often marginal (Thangaraju *et al*, 1994; Love *et al*, 1994; Decensi *et al*, 1998; Vrbanec *et al*, 1998). Tamoxifen has also been shown to lower Lp(a) levels (Decensi *et al*, 1998). Triglyceride levels appear to increase (Dziewulska-Bokiniec *et al*, 1994; Grey *et al*, 1995). Levels of apolipoprotein B-100 and apolipoprotein A-I are also reduced with tamoxifen (Elisaf *et al*, 1996). These effects are thought to be caused by the ability of tamoxifen to act as a partial agonist in some tissues. By contrast, the aromatase inhibitors do not have oestrogenic effects, which has led to concern about possible detrimental effects to lipid profiles.

### Aromatase inhibitors

To date, few studies have been conducted that include an assessment of lipid effects, and conflicting results have been obtained from those that have. The different aromatase inhibitors appear to have different effects on lipid profiles (Table 1). This may be a result of their different modes of action. Anastrozole and letrozole are nonsteroidal aromatase inhibitors, while exemestane is a steroidal aromatase inhibitor. The steroidal inhibitors are analogues of androstenedione and bind to the same site on the aromatase molecule, but unlike androstenedione they bind irreversibly, because of their conversion to reactive intermediates by aromatase. The nonsteroidal agents, by contrast, bind reversibly to the heme group of the enzyme.

#### Anastrozole

In several small studies, anastrozole showed no marked effects on lipid profile (Dewar *et al*, 2000; Kataja *et al*, 2002; Sawada and Sato, 2003; Wojtacki *et al*, 2004). Kataja *et al*, (2002) showed LDL levels fell with both exemestane and anastrozole and triglycerides fell with exemestane, but remained stable with anastrozole. However, anastrozole was associated with a higher incidence of hypercholesterolaemia than tamoxifen in the ATAC study (ATAC Trialists Group, 2002). In the Italian Tamoxifen Arimidex (ITA) trial, patients switching to anastrozole after 2 or more years of tamoxifen were also found to have higher levels of hypercholesterolaemia than those continuing on tamoxifen, 8.1 and 2.7%, respectively (Boccardo *et al*, 2003). In addition, a recent study of the effect of anastrozole on serum lipid profiles in 38 postmenopausal patients with breast cancer found significant increases in total cholesterol, LDL and HDL cholesterol, apolipoprotein A1, B and lp(a) (Hozumi *et al*, 2004). Anastrozole was associated with a slightly higher incidence of ischaemic cardiovascular disease than tamoxifen in the ATAC study, but this was not significant (ATAC Trialists Group, 2002).

#### Letrozole

Increases in total serum cholesterol, LDL cholesterol, apo B and serum-lipid risk ratios for cardiovascular disease were found in some studies (Nicolaides *et al*, 2000; Elisaf *et al*, 2001), while others have found no changes in lipid profile (Harper-Wynne *et al*, 2001). In the first interim analysis of a 5-year study of letrozole therapy after the completion of 5 years’ tamoxifen treatment, Goss *et al* (2003) found no significant differences in the rates of cardiovascular events between the letrozole group (4.1%) and the placebo group (3.6%), and there were no reports of drug-related hypercholesterolaemia. By contrast, the first results of the BIG 1-98 trial comparing letrozole with tamoxifen have shown that 43.6% of letrozole-treated patients developed mild to moderate hypercholesterolaemia *vs* 18.2% of tamoxifen-treated patients (Thurlimann, 2005). While more tamoxifen-treated patients suffered thromboembolic events than letrozole patients (grades 3–5, 2 *vs* 0.8%), a higher incidence of cardiovascular events was seen with letrozole-treated patients than tamoxifen patients (grades 3–5, 3.6 *vs* 2.5%), although this was not significant. Longer-term follow-up will be required to establish the significance of hypercholesterolaemia seen in the BIG 1–98 trial (Thurlimann, 2005).

#### Exemestane

The lipid effects of exemestane have, perhaps, been more closely studied than those of the other aromatase inhibitors. In animal studies, exemestane reversed the increase in LDL cholesterol and total cholesterol seen in ovariectomized cycling Sprague–Dawley rats (Goss *et al*, 2001).

In a 3-month study compared with anastrozole and tamoxifen in postmenopausal women with breast cancer (*n*=30), exemestane produced a small decrease in serum cholesterol and LDL cholesterol, decreases in triglycerides and HDL cholesterol (Kataja *et al*, 2002). In the same study, anastrozole showed similar results except for a small increase in HDL cholesterol (Kataja *et al*, 2002). The EORTC trial 10951 randomized 382 postmenopausal breast cancer patients to exemestane or tamoxifen once daily as first-line treatment in the metastatic setting (Atalay *et al*, 2004; Paridaens *et al*, 2004). A companion substudy to this trial analysing effects on lipid profiles revealed no adverse effects on total cholesterol, HDL cholesterol, apolipoprotein A1 and B or Lp(a) levels at 8, 24 and 48 weeks of treatment with either exemestane or tamoxifen (72 patients included in the statistical analysis) (Atalay *et al*, 2004). By contrast, exemestane decreased but tamoxifen increased triglyceride levels. In the Greek substudy of the TEAM trial, which is comparing tamoxifen with exemestane as initial therapy, baseline lipid levels have been compared with levels at 3 and 6 months of treatment in 37 patients. At 6 months, exemestane appeared to stabilize total cholesterol and HDL cholesterol levels as did tamoxifen (Markopoulos *et al*, 2003). Exemestane had a nonsignificant trend to increase LDL cholesterol levels at 3 and 6 months, but reduced triglyceride levels at both time points and by about 10% at 6 months (Markopoulos *et al*, 2003). Finally, although cholesterol levels were not systematically measured in the recent IES study comparing exemestane with tamoxifen in 4742 women, it was noted that there was no significant difference in the incidence of myocardial infarction between the two groups (Coombes *et al*, 2004).

With regard to postmenopausal patients with early breast cancer, results were reported at the 2004 ASCO meeting of the effects of exemestane, 25 mg day^−1^, *vs* placebo on lipid and coagulation profiles (Krag *et al*, 2004). After 2 years, lipid profiles were similar for exemestane and placebo with decreased levels of cholesterol, LDL cholesterol, triglycerides, apolipoprotein A1 and B and Lp(a) seen in both groups. The only difference was a small decrease in HDL cholesterol seen with exemestane only (Krag *et al*, 2004).

## CLINICAL SIGNIFICANCE OF LIPID EFFECTS

The significance of changes in lipid profiles seen with different aromatase inhibitors is impossible to assess from the limited data available. The changes seen are generally small. For example, in the study by Elisaf *et al*, (2001), mean LDL cholesterol levels increased from 148±50 to 170±53 mg dl^−1^ after 16 weeks with letrozole, while the total cholesterol to HDL cholesterol ratio increased from 3.94±1.46 to 4.48±1.40 after 16 weeks. Such a change might be sufficient to alter a patient's risk category from low to moderate depending on other risk factors, therefore triggering the need for lipid-lowering treatment.

The benefits of receiving an aromatase inhibitor are likely to outweigh the disadvantages of any changes to lipid profiles, particularly for patients with advanced disease. However, when aromatase inhibitors are used in early disease or in the prevention setting, an increase of 10–15% in circulating cholesterol and triglycerides may have a significant impact on the risk of cardiovascular disease, and monitoring of blood lipid levels and instigation of lipid lowering treatment if required should be undertaken. In such instances, the choice of agent may be particularly important in the adjuvant and prevention settings, and agents that have minimal effects on lipids will be preferred, especially if evidence of reduced cardiovascular morbidity can be demonstrated in clinical trials.

## EFFECTS OF ANTI-OESTROGEN TREATMENT ON STROKE AND THROMBOEMBOLISM

Tamoxifen increases the risk of venous thromboembolism, but it has been unclear as to the implications of this for risk of stroke. However, a recently published meta-analysis of tamoxifen breast cancer trials revealed an increased risk of stroke with tamoxifen compared with placebo/control (Bushnell and Goldstein, 2004). Results of the meta-analysis showed tamoxifen-treated patients had an 82% and a 29% increased risk of ischaemic stroke and any stroke, respectively, although the authors noted that the absolute risk was small (Bushnell and Goldstein, 2004). The notable increased risk in ischaemic stroke was suggested to be a consequence of the tamoxifen-associated increase in thrombosis. Further studies with tamoxifen to include measurements of prespecified cerebrovascular outcomes will help to clarify the stroke risk with tamoxifen; meanwhile, tamoxifen may be best avoided for women with a history of stroke or thrombosis.

The risk of cerebrovascular and thromboembolic events has been seen to be significantly higher with tamoxifen than the aromatase inhibitors; for example, anastrozole was associated with significantly fewer ischaemic cerebrovascular events (*P*=0.0006) and thromboembolic events (*P*=0.0006) than tamoxifen in the ATAC trial (ATAC Trialists Group, 2002). Results of the IES study also showed thromboembolic events as more frequent with tamoxifen than exemestane (*P*=0.007) (Coombes *et al*, 2004). In addition, no thromboembolic events were observed with letrozole at a dose of 2.5 mg in a study compared with megestrol acetate for advanced breast cancer (Table 2) (Dombernowsky *et al*, 1998). Since the aromatase inhibitors are not usually associated with thrombosis, women with a previous history of thrombosis or stroke should receive an aromatase inhibitor in preference to tamoxifen.

## ONGOING AND PLANNED CLINICAL TRIALS

Clearly, more data are needed before the relevance of the changes in lipid levels with aromatase inhibitors on cardiovascular morbidity can be determined. Indeed, most of the ongoing clinical trials are including analysis of lipid effects as part of their protocol. However, care needs to be taken when evaluating lipid effects in studies comparing aromatase inhibitors with tamoxifen, because tamoxifen itself influences lipid values.

Among the ongoing trials that will report on lipid changes are:

- MA17L: is a substudy of the MA17 study involving 300 patients randomised to letrozole or placebo after completing 5 years with tamoxifen.
- BIG FEMTA(1-98): is comparing 5 years of tamoxifen, 5 years of letrozole or sequenced therapy of 2–3 years each starting with either tamoxifen or letrozole.
- NSABP B33: is a randomised, placebo-controlled, double-blind trial evaluating the effect of exemestane in 3000 clinical stage T1-3 N0-1 M0 postmenopausal breast cancer patients completing at least 5 years of tamoxifen therapy.
- TEAM: preliminary lipid results have already been reported from this phase III randomised study of adjuvant exemestane *vs* adjuvant tamoxifen in 4400 postmenopausal women with early breast cancer (Markopoulos *et al*, 2003). Final results are awaited.
- ARNO: in this study, women receive 2 years of tamoxifen followed by randomisation to 3 years of either anastrozole or tamoxifen.
- ICCG: in this study, women receive 3 years of tamoxifen followed by 2 years of either tamoxifen or exemestane.
- IMPACT: compares anastrozole *vs* tamoxifen alone and in combination as neoadjuvant treatment of oestrogen receptor positive operable breast cancer in postmenopausal women.
- NCIC CTG MAP2: is a randomised study of the effect of exemestane *vs* placebo on breast density in postmenopausal women at increased risk for development of breast cancer.

## CONCLUSIONS

Concern about lipid changes associated with aromatase inhibitors leading to increased cardiovascular deaths has not so far been borne out in the limited data from adjuvant trials. Of the available data on effects of aromatase inhibitors on serum lipids from short-term studies, exemestane has little or possibly a slight beneficial effect on serum lipids, and anastrozole appears to have little effect or possibly an adverse effect, while letrozole may have a detrimental effect.

## Figures and Tables

**Table 1:** Effects of anti-oestrogen therapy on plasma lipid levels (Dewar *et al*, 2000; Nicolaides *et al*, 2000; Elisaf *et al*, 2001; Goss *et al*, 2001, 2003; Harper-Wynne *et al*, 2001; ATAC, 2002; Kataja *et al*, 2002; Boccardo *et al*, 2003; Markopoulos *et al*, 2003; Sawada and Sato, 2003; Atalay *et al*, 2004; Coombes *et al*, 2004; Hozumi *et al*, 2004; Paridaens *et al*, 2004; Wojtacki *et al*, 2004)

	**LDL cholesterol**	**HDL cholesterol**	**Total cholesterol**	**Triglycerides**	**Total : HDL cholesterol**	**Lp(a)**	**Apo B**
Tamoxifen	↓	−	↓	?	?	↓	↓
Anastrozole	↑−	↑/−	↑/−	−	−	↑/−	↑/−
Letrozole	↑/−	−	↑/−	−	↑/−	?	↑/−
Exemestane	↓−	↓/−	↓/−	↓	?	−	−

Key: ↑ =increased; ↓=decreased; −=no change; ?=unknown.

**Table 2:** Incidence of thromboembolic events with aromatase inhibitors and comparators

	**Thromboembolic events (% of patients)**		
**Reference**	**Aromatase inhibitor**	**Comparator**	***P*-value**
ATAC (2002)	Anastrozole 64 (2.1%)	Tamoxifen 109 (3.5%)	=0.0006
Coombes *et al* (2004) (IES Trial)	Exemestane 30 (1.3%)	Tamoxifen 55 (2.4%)	=0.007
Dombernowsky *et al* (1998)	Letrozole (2.5 mg) 1 (0%)	Megestrol acetate 15 (7.9%)	Unknown
	Letrozole (0.5 mg) 2 (1%)		
Goss *et al* (2003) (MA-17 trial) (cardiovascular events)	Letrozole (2.5 mg) 88 (4.1%)	Placebo 77 (3.6%)	=0.4

## References

[bib1] ATAC (Arimidex, Tamoxifen Alone or in Combination) Trialists' Group (2002) Anastrozole alone or in combination with tamoxifen *vs* tamoxifen alone for adjuvant treatment of postmenopausal women with early breast cancer: first results of the ATAC randomised trial. Lancet 359: 2131–2139 12090977 10.1016/s0140-6736(02)09088-8

[bib2] Atalay G, Dirix L, Biganzoli L, Beex L, Nooij M, Cameron D, Lohrisch C, Cufer T, Lobelle JP, Mattiaci MR, Piccart M, Paridaens R (2004) The effect of exemestane on serum lipid profile in postmenopausal women with metastatic breast cancer: a companion study to EORTC Trial 10951, ‘Randomized phase II study in first line hormonal treatment for metastatic breast cancer with exemestane or tamoxifen in postmenopausal patients’. Ann Oncol 15: 211–217 14760111 10.1093/annonc/mdh064

[bib3] Boccardo F, Rubagotti A, Amoroso D, Mesiti M, Massobrio M, Benedetto C, Porpiglia M, Rinaldini M, Paladini G, Distante V, Franchi R, Failla G, Bordonaro R, Sismondi P, on Behalf of the Italian Tamoxifen Arimidex (ITA) Trial (2003) Anastrozole appears to be superior to tamoxifen in women already receiving adjuvant tamoxifen in women already receiving adjuvant tamoxifen treatment. 26th Annual San Antonio Breast Cancer Symposium; 3–6 December 2003: San Antonio, Texas. Abstract 3

[bib4] Bushnell CD, Goldstein LB (2004) Risk of ischemic stroke with tamoxifen treatment for breast cancer. A meta-analysis. Neurology 63: 1230–1233 15477543 10.1212/01.wnl.0000140491.54664.50

[bib5] Campos H, McNamara JR, Wilson PW, Ordovas JM, Schaefer EJ (1988) Differences in low density lipoprotein subfractions and apolipoproteins in premenopausal and postmenopausal women. J Clin Endocrinol Metab 67: 30–35 3379134 10.1210/jcem-67-1-30

[bib6] Coombes RC, Hall E, Gibson LJ, Paridaens R, Jassem J, Delozier T, Jones SE, Alvarez I, Bertelli G, Ortmann O, Coates AS, Bajetta E, Dodwell D, Coleman RE, Fallowfield LJ, Mickiewicz E, Andersen J, Lonning PE, Cocconi G, Stewart A, Stuart N, Snowdon CF, Carpentieri M, Massimini G, Bliss JM, Intergroup Exemestane Study (2004) A randomized trial of exemestane after two to three years of tamoxifen therapy in postmenopausal women with primary breast cancer. N Engl J Med 350: 1081–1092 15014181 10.1056/NEJMoa040331

[bib7] Cushman M, Costantino JP, Tracy RP, Song K, Buckley L, Roberts JD, Krag DN (2001) Tamoxifen and cardiac risk factors in healthy women. Suggestion of an anti-inflammatory effect. Arterioscler Thromb Vasc Biol 21: 255–261 11156862 10.1161/01.atv.21.2.255

[bib8] Danesh J, Collins R, Peto R (2000) Lipoprotein(a) and coronary heart disease. Meta-analysis of prospective studies. Circulation 102: 1082–1085 10973834 10.1161/01.cir.102.10.1082

[bib9] de Aloysio D, Gambacciani M, Meschia M, Pansini F, Bacchi Modena A, Bolis PF, Massobrio M, Maiocchi G, Peruzzi E (1999) The effect of menopause on blood lipid and lipoprotein levels. The Icarus Study Group. Atherosclerosis 147: 147–153 10525136 10.1016/s0021-9150(99)00315-9

[bib10] Decensi A, Bonanni B, Guerrieri-Gonzaga A, Gandini S, Robertson C, Johansson H, Travaglini R, Sandri MT, Tessadrelli A, Farante G, Salinaro F, Bettega D, Barreca A, Boyle P, Costa A, Veronesi U (1998) Biologic activity of tamoxifen at low doses in healthy women. J Natl Cancer Inst 90: 1461–1467 9776411 10.1093/jnci/90.19.1461

[bib11] Dewar J, Nabholtz JM, Bonneterre J, Buzdar A, Robertson JFR, Thurlimann B, Clark G (2000) The effect of anastrozole (Arimidex) on plasma lipids – data from a randomised comparison of anastrozole *vs* tamoxifen in postmenopausal women with advanced breast cancer. Breast Cancer Res Treat 64: 51 (Abstract 164)

[bib12] Do KA, Green A, Guthrie JR, Dudley EC, Burger HG, Dennerstein L (2000) Longitudinal study of risk factors for coronary heart disease across the menopausal transition. Am J Epidemiol 151: 584–593 10733040 10.1093/oxfordjournals.aje.a010246

[bib13] Dombernowsky P, Smith I, Falkson G, Leonard R, Panasci L, Bellmunt J, Bezwoda W, Gardin G, Gudgeon A, Morgan M, Fornasiero A, Hoffmann W, Michel J, Hatschek T, Tjabbes T, Chaudri HA, Hornberger U, Trunet PF (1998) Letrozole, a new oral aromatase inhibitor for advanced breast cancer: double-blind randomized trial showing a dose effect and improved efficacy and tolerability compared with megestrol acetate. J Clin Oncol 16: 453–461 9469328 10.1200/JCO.1998.16.2.453

[bib14] Dziewulska-Bokiniec A, Wojtacki J, Skokowski J, Kortas B (1994) The effect of tamoxifen treatment on serum cholesterol fractions in breast cancer women. Neoplasma 41: 13–16 8202188

[bib15] Elisaf M, Bairaktari E, Nicolaides C, Fountzilas G, Tzallas C, Siamopoulos K, Tsolas O, Pavlidis N (1996) The beneficial effect of tamoxifen on serum lipoprotein-A levels: an additional anti-atherogenic property. Anticancer Res 16: 2725–2728 8917378

[bib16] Elisaf MS, Bairaktari ET, Nicolaides C, Kakaidi B, Tzallas CS, Katsaraki A, Pavlidis NA (2001) Effect of letrozole on the lipid profile in postmenopausal women with breast cancer. Eur J Cancer 37: 1510–1513 11506958 10.1016/s0959-8049(01)00155-1

[bib17] Goss PE, Grynpas MD, Josse R (2001) The effects of the steroidal aromatase inactivator exemestane on bone and lipid metabolism in the ovariectomized rat. Breast Cancer Res Treat 69: 224 (Abstract 132)

[bib18] Goss PE, Ingle JN, Martino S, Robert NJ, Muss HB, Piccart MJ, Castiglione M, Tu D, Shepherd LE, Pritchard KI, Livingston RB, Davidson NE, Norton L, Perez EA, Abrams JS, Therasse P, Palmer MJ, Pater JL (2003) A randomized trial of letrozole in postmenopausal women after five years of tamoxifen therapy for early-stage breast cancer. N Engl J Med 349: 1793–1802 14551341 10.1056/NEJMoa032312

[bib19] Grey AB, Stapleton JP, Evans MC, Reid IR (1995) The effect of the anti-estrogen tamoxifen on cardiovascular risk factors in normal postmenopausal women. J Clin Endocrinol Metab 80: 3191–3195 7593425 10.1210/jcem.80.11.7593425

[bib20] Harper-Wynne C, Ross G, Sacks N, Dowsett M (2001) Effects of the aromatase inhibitor letrozole on breast cell proliferation and bone/lipid indices in healthy postmenopausal women: pilot prevention study. Breast Cancer Res Treat 69: 225 (Abstract 136)

[bib21] Hozumi Y, Saito T, Inoue K, Shiozawa M, Omoto Y, Tabei T, Nagai H (2004) Effects of anastrozole on the lipid profile in postmenopausal breast cancer patients – a preliminary study. Fourth European Breast Cancer Conference; 16–20 March 2004; Hamburg, Germany. Abstract 300

[bib22] Hulley S, Grady D, Bush T, Furberg C, Herrington D, Riggs B, Vittinghoff E (1998) Randomized trial of estrogen plus progestin for secondary prevention of coronary heart disease in postmenopausal women. JAMA 280: 605–613 9718051 10.1001/jama.280.7.605

[bib23] Kannel WB (1987) Metabolic risk factors for coronary heart disease in women: perspective from the Framingham Study. Am Heart J 114: 413–419 3604900 10.1016/0002-8703(87)90511-4

[bib24] Kataja V, Hietanen P, Joensuu H, Ala-Luhtala T, Asola R, Kokko R, Holli K (2002) The effects of adjuvant anastrozole, exemestane, tamoxifen, and toremifene on serum lipids in postmenopausal women with breast cancer – a randomised study. 25th San Antonio Breast Cancer Symposium; 11–14 December 2002; San Antonio, Texas. Abstract 634

[bib25] Korhonen T, Savolainen MJ, Ikäheimo M, Linnaluoto MK, Kervinen K, Kesäniemi YA (1996) Association of lipoprotein cholesterol and triglycerides with the severity of coronary artery disease in men and women. Atherosclerosis 127: 213–220 9125311 10.1016/s0021-9150(96)05958-8

[bib26] Krag LE, Geisler J, Lonning PE, Ottestad L, Risberg T, Hagen AI, Lien EA, Polli A, Paolini J, Massimini G (2004) Lipid and coagulation profile in postmenopausal women with early breast cancer at low risk treated with exemestane: a randomised, placebo-controlled study. American Society of Clinical Oncology; 5–8 June 2004; New Orleans, USA. Abstract 650

[bib27] La Rosa JC (1997) Triglycerides and coronary risk in women and the elderly. Arch Intern Med 157: 961–968 9140266

[bib28] Levi F, Randimbison L, Te VC, La Vecchia C (2002) Long-term mortality of women with a diagnosis of breast cancer. Oncology 63: 266–269 12381906 10.1159/000065475

[bib29] Love RR, Wiebe DA, Feyzi JM, Newcomb PA, Chappell RJ (1994) Effects of tamoxifen on cardiovascular risk factors in postmenopausal women after 5 years of treatment. J Natl Cancer Inst 86: 1534–1539 7932809 10.1093/jnci/86.20.1534

[bib30] Markopoulos C, Polychronis A, Fafarelos C, Zobolas V, Bafaloukos D, Papadiamantis J (2003) The effect of exemestane (Aromasin®) on the lipidemic profile of breast cancer patients: preliminary results of the TEAM trial Greek sub-study. 26th Annual San Antonio Breast Cancer Symposium, 3–6 December 2003; San Antonio, Texas. Abstract 440

[bib31] Matthews KA, Wing RR, Kuller LH, Meilahn EN, Plantinga P (1994) Influence of the perimenopause on cardiovascular risk factors and symptoms of middle-aged healthy women. Arch Intern Med 154: 2349–2355 7944857

[bib32] Miller-Bass K, Newschaffer CJ, Klag MJ, Bush TL (1993) Plasma lipoprotein levels as predictors of cardiovascular death in women. Arch Intern Med 153: 2209–2216 8215724

[bib33] Nicolaides C, Elisaf M, Bairaktari E, Kakaidi V, Katsaraki A, Pavlidis N (2000) The effect of the aromatase inhibitor letrozole on lipid parameters in postmenopausal women with breast cancer. A preliminary report. Ann Oncol 11(Suppl 4): 12 (Abstract 38 O)

[bib34] Paridaens R, Therasse P, Dirix L, Beex L, Piccart MJ, Cameron D, Cufer T, Roozendael K, Nooy M, Mattiacci MR (2004) First results of a randomised phase III trial comparing exemestane *vs* tamoxifen as first-line hormone therapy (HT) for postmenopausal women with metastatic breast cancer (MBC) – EORTC 10951 in collaboration with the exemestane working group and NCIC. Fourth European Breast Cancer Conference; 16–20 March 2004; Hamburg, Germany. Abstract 241

[bib35] Sawada S, Sato K (2003) Effect of anastrozole and tamoxifen on serum lipid levels in Japanese postmenopausal women with early breast cancer. 26th San Antonio Breast Cancer Symposium; 3–6 December 2003; San Antonio, Texas, Abstract 143

[bib36] Schaefer EJ, Lamon-Fava S, Cohn SD, Schaefer MM, Ordovas JM, Castelli WP, Wilson PW (1994) Effects of age, gender, and menopausal status on plasma low density lipoprotein cholesterol and apolipoprotein B levels in the Framingham Offspring Study. J Lipid Res 35: 779–792 8071601

[bib37] Stevenson JC, Crook D, Godsland IF (1993) Influence of age and menopause on serum lipids and lipoproteins in healthy women. Atherosclerosis 98: 83–90 8457253 10.1016/0021-9150(93)90225-j

[bib38] Thangaraju M, Kumar K, Gandhirajan R, Sachdanandam P (1994) Effect of tamoxifen on plasma lipids and lipoproteins in postmenopausal women with breast cancer. Cancer 73: 659–663 8299087 10.1002/1097-0142(19940201)73:3<659::aid-cncr2820730325>3.0.co;2-h

[bib39] Thurlimann B (2005) Letrozole as adjuvant endocrine therapy for postmenopausal women with receptor-positive breast cancer. First results of IBCSG 18-98/BIG 1-98. Presented at the Ninth International Conference of Primary Therapy of Early Breast Cancer; 26–29 January 2005; St Gallen, Switzerland

[bib40] Tremollieres FA, Pouilles JM, Cauneille C, Ribot C (1999) Coronary heart disease risk factors and menopause: a study in 1684 French women. Atherosclerosis 142: 415–423 10030394 10.1016/s0021-9150(98)00252-4

[bib41] Vrbanec D, Reiner Z, Belev B, Plestina S (1998) Changes in serum lipid and lipoprotein levels in postmenopausal patients with node-positive breast cancer treated with tamoxifen. Tumori 84: 687–690 10080678 10.1177/030089169808400615

[bib42] Wojtacki J, Lesniewski-Kmak K, Pawlack W, Nowicka E (2004) Anastrozole therapy and lipid profile: an update. Fourth European Breast Cancer Conference; 16–20 March 2004; Hamburg, Germany. Abstract 297

[bib43] Women's Health Initiative Steering Committee (2004) Effects of conjugated equine estrogen in postmenopausal women with hysterectomy. The Women's Health Initiative randomized controlled trial. JAMA 291: 1701–1712 15082697 10.1001/jama.291.14.1701

